# Properties and use of novel replication-competent vectors based on Semliki Forest virus

**DOI:** 10.1186/1743-422X-6-33

**Published:** 2009-03-24

**Authors:** Kai Rausalu, Anna Iofik, Liane Ülper, Liis Karo-Astover, Valeria Lulla, Andres Merits

**Affiliations:** 1 Institute of Technology, University of Tartu, Nooruse 1, 50411, Tartu, Estonia

## Abstract

**Background:**

Semliki Forest virus (SFV) has a positive strand RNA genome and infects different cells of vertebrates and invertebrates. The 5' two-thirds of the genome encodes non-structural proteins that are required for virus replication and synthesis of subgenomic (SG) mRNA for structural proteins. SG-mRNA is generated by internal initiation at the SG-promoter that is located at the complementary minus-strand template. Different types of expression systems including replication-competent vectors, which represent alphavirus genomes with inserted expression units, have been developed. The replication-competent vectors represent useful tools for studying alphaviruses and have potential therapeutic applications. In both cases, the properties of the vector, such as its genetic stability and expression level of the protein of interest, are important.

**Results:**

We analysed 14 candidates of replication-competent vectors based on the genome of an SFV4 isolate that contained a duplicated SG promoter or an internal ribosomal entry site (IRES)-element controlled marker gene. It was found that the IRES elements and the minimal -21 to +5 SG promoter were non-functional in the context of these vectors. The efficient SG promoters contained at least 26 residues upstream of the start site of SG mRNA. The insertion site of the SG promoter and its length affected the genetic stability of the vectors, which was always higher when the SG promoter was inserted downstream of the coding region for structural proteins. The stability also depended on the conditions used for vector propagation. A procedure based on the *in vitro *transcription of ligation products was used for generation of replication-competent vector-based expression libraries that contained hundreds of thousands of different genomes, and maintained genetic diversity and the ability to express inserted genes over five passages in cell culture.

**Conclusion:**

The properties of replication-competent vectors of alphaviruses depend on the details of their construction. In the case of SFV4, such vectors should contain the SG promoter with structural characteristics for this isolate. The main factor for instability of SFV4-based replication-competent vectors was the deletion of genes of interest, since the resulting shorter genomes had a growth advantage over the original vector.

## Background

The alphavirus (family *Togaviridae*) genome is a positive-stranded RNA that is approximately 11.5 kb in length. It encodes two large polyprotein precursors that are co- and post-translationally processed into active processing intermediates and mature proteins [1]. The structural proteins, encoded by the 3' one-third of the genome, are translated from a subgenomic (SG) mRNA, which is generated by internal initiation from the SG promoter that is located on the complementary minus-strand template. The non-structural (ns) polyprotein is translated directly from the viral genomic RNA. It is processed into individual components, the ns proteins nsP1–nsP4. The nsPs have multiple enzymatic and non-enzymatic functions required in viral RNA replication [2]. Semliki Forest virus (SFV), Sindbis virus (SINV) and Venezuelan equine encephalitis virus (VEEV) are the best studied alphaviruses, and have also been used for the development of gene expression systems [3-5].

Alphavirus-based expression vectors have been classified into three groups: virus-like particles (VLPs), layered DNA-RNA vectors, and replication-competent vectors [6]. VLPs are produced by co-transfection of cells with an *in-vitro-*transcribed replicon RNA, which represents the alphavirus genome in which the region coding for structural proteins has been replaced by a gene of interest and a helper RNA encoding for structural proteins [3,7]. In VLP-infected cells, a high level of synthesis of foreign proteins takes place, and at the same time, the system is self-limiting because helper-RNAs are not encapsidated. Layered DNA-RNA vectors represent systems where the cDNA of a replicon vector is flanked with eukaryotic transcription elements, with a promoter at the 5'-end and a poly(A) signal at the 3' end. Layered systems can be used for VLP production; however, more often, they are used for rescue of alphavirus replicons and subsequent protein expression in transfected cells [8-11]. Layered systems have been used to rescue alphavirus genomes in transfected cells [12,13], but so far, not for construction and rescue of replication-competent alphavirus-based vectors.

The replication-competent alphavirus vectors are designed to undergo several rounds of multiplication in cell culture or in hosts. Several approaches have been used for construction of such vectors. First, foreign gene or genes can be inserted in selected position(s) in ns-polyprotein in a way that it is expressed in form of a fusion-protein together with some alphavirus ns-protein [14-16]. Such vectors express the foreign protein at early stages of infection. Second, a protein can also be expressed as cleavable part of an alphavirus-encoded structural or ns polyprotein [17-19]. Third, the number of expression units in a vector can be increased by inserting duplicated copies of SG promoters into the alphavirus genome, either in the 3'-untranslated region (UTR) or into the short intergenic region between the ns and structural regions [20-23]. Such vectors have served as useful instruments for analysis of the structure, function and evolution of SG promoters [20,24-27] and for the analysis of viral movement in infected organisms [28]. Some of these vectors have been used as tools for foreign protein expression [21]. Replication-competent vectors have also been used for cloning and analysis of libraries generated by random or insertion mutagenesis [15,16,25-27], and the use of such vectors for therapeutic and prophylactic applications has also been proposed [6]. In addition to the duplication of SG promoters, the expression of a foreign gene by using an internal ribosomal entry site (IRES) has been described for alphavirus VLPs [29], DNA-RNA layered vectors [30], and replication-competent vectors based on rubella virus, another member of the *Togaviridae *family [31]. The IRES element has also been utilized to develop VEEV variants incapable of replication in mosquito cells [32].

The increase in the genome size of the vector caused by duplications of viral sequences and/or gene insertion slows down its replication, which can cause problems with regulation of gene expression and packaging of larger-than-normal genomes into icosahedral capsids. Therefore, the intrinsic problem for such vectors is their genetic instability, which results from the lack of proofreading ability of alphavirus RNA polymerases and spontaneous recombination in the vector genome [17-19,23,31,33]. It has been reported that the site and mode of the marker gene insertion [15,16,18], the positioning of duplicated SG promoter [23], the host and conditions used for vector propagation [19,23], as well as the nature of the inserted gene itself (our unpublished observations) all affect the stability of replication-competent alphavirus vectors.

Expression of the protein of interest as part of a structural or ns polyprotein strictly links the time and level of its expression with those of the corresponding viral proteins. In addition, the requirement to maintain the reading frames of the structural or ns polyproteins complicates the cloning procedure and severely hampers the usage of such vectors for cloning expression libraries. These problems do not exist for vectors with duplicated SG promoters such as the SFV-based vector VA7. Despite its low genetic stability, this vector has been successfully used for various applications [22,34-36].

For construction of efficient and stable vectors with duplicated SG promoters, information about the structure and function of this element is essential. The SG promoter of SINV has been studied extensively for almost two decades. Its minimal sequence consists of 24 residues located from -19 to +5 with respect of the start site of SG mRNA (hereafter, SG promoters are designated as 19/5 etc.), and it is approximately 3–6-fold less active than a full-size SG promoter (98/14 or 40/14) of SINV [20,27,37]. In addition, molecular interactions between the replicase of SINV and its SG promoter sequences have been studied [38-40], and specific mechanisms involved in the initiation of translation of SG mRNA have been analysed [41-43]. Much less is known about the SG promoter of SFV. It has been reported that the minimal SG promoter of some SFV isolates (sequence published in [44]), but not that of isolate SFV4 (sequence published in [45]), is active in the context of replication-competent vectors of SINV. This difference has been attributed to the unusual G residue at the -1 position of the SG promoter of SFV4 (most alphaviruses have an A residue at this position). It has been hypothesized that, in the context of the native genome of SFV4, the effect of the -1 G residue might be compensated by some other changes [24].

The aim of the present study was to obtain genetically stable SFV4-based replication-competent vectors that can also be used for construction of expression libraries. It was found that the SFV4 vectors with a duplicated SG promoter placed at the 3'-UTR were invariably more stable than those containing similar duplications in the intergenic region. The inserted IRES elements were found to be non-functional in the context of replication-competent SFV4 vectors. A novel approach, based on *in vitro *transcription of ligation products, which allowed conversion of a replicon vector into a replication-competent vector and cloning of expression libraries, was developed.

## Results

### Construction and viability of SFV4-based replication-competent vectors with duplicated SG promoters

We have previously observed, using the SFV-based VLP system, that expression of the protein of interest from a short 20/6 SG promoter of SFV4 was considerably weaker than that from different IRES elements [29]. This indicates that the minimal SG promoter for SFV4 is likely to be longer than the minimal 19/5 SG promoter for SINV. Therefore, two sets of vectors with duplicated SG promoters, each consisting of four different constructs, were made and tested for their ability to express SFV4 ns proteins (exemplified by nsP1), structural proteins (exemplified by capsid protein) and inserted marker protein (destabilized EGFP, d1EGFP).

**Figure 1:**
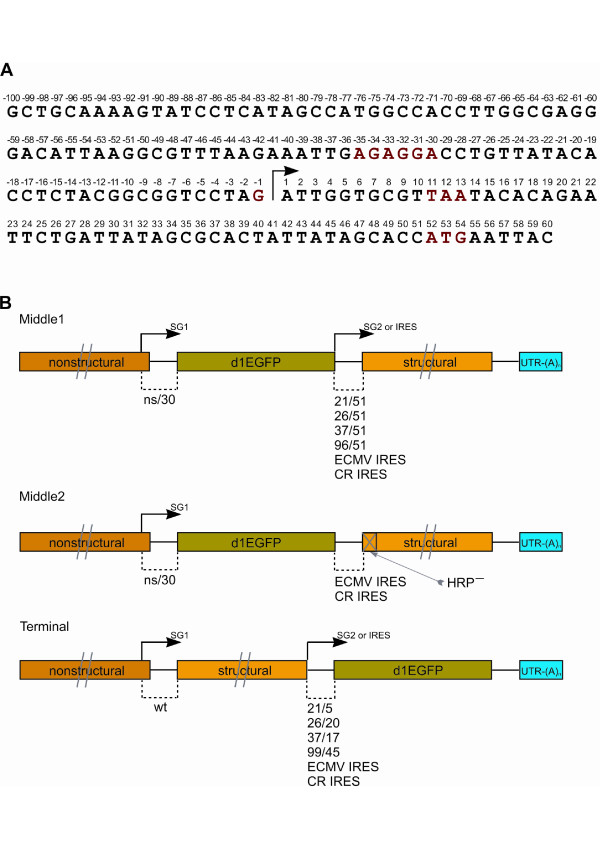
**Sequence of SG promoter region and schematic presentation of SFV4-based replication-competent vectors**. (A) Sequence of SG region of SFV4 (in positive strand) from position -100 to 60. Arrow indicates start site of SG mRNA, short conserved sequence element of SG promoters of alphaviruses (-35 to -30), unique -1 G residue of SG promoter of SFV4, termination codon for ns-polyprotein reading frame (11 to 13), and initiation codon for structural polyprotein (52 to 54) are shown in color. (B) Schematic presentation of the genomes of replication-competent SFV4-based vectors. "Middle1" represents genomes of middle vectors rescued by use of infectious *in vitro *transcripts and "Middle2" represents genomes of middle vectors rescued from infectious plasmid by cellular RNA synthesis and splicing machinery. Arrows indicate SG promoters, and UTR-(A)_n _indicates 3'-UTR of SFV4 with poly(A) sequence. The structure of SG promoters is shown below the drawing, "wt" indicates the native SG promoter of SFV4 and "ns/30" indicates the native SG promoter with truncated downstream region (residues 31 to 51 are deleted). HRP^- ^on structural protein indicates the destabilized capsid hairpin element.

Terminal (T) vectors were designated as SFV4-T21/5-d1EGFP, SFV4-T26/20-d1EGFP, SFV4-T37/17-d1EGFP and SFV4-T99/45-d1EGFP. They contained the indicated SG promoter and d1EGFP insertion immediately downstream of the termination codon for E1 protein (Figure 1A and 1B). Middle (M) vectors were designated as SFV4-d1EGFP-M96, SFV4-d1EGFP-M37, SFV4-d1EGFP-M26 and SFV4-d1EGFP-M21. In the middle vectors, the expression of inserted d1EGFP marker was derived from native ns/30 SG promoter of SFV4, while the expression of viral structural proteins was achieved by a duplicated SG promoter (Figure 1A and 1B). All duplicated SG promoters in middle vectors were designed to produce SG mRNA with a full-length (51 b) leader sequence, since it has been reported that extensive deletions in the 3'-region of the SG mRNA leader sequence of SINV reduce the translation of downstream regions by two-fold or more [42]. With that exception, the two sets of vectors contained duplicated SG promoters of comparable composition.

**Figure 2:**
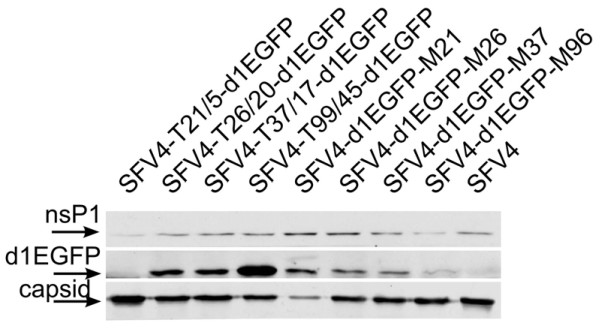
**Protein expression in BHK-21 cells transfected with replication-competent vectors with duplicated SG promoters**. BHK-21 cells were transfected with equal amount of *in vitro *transcripts prepared from pSFV4-T21/5-d1EGFP, pSFV4-T26/20-d1EGFP, pSFV4-T37/17-d1EGFP, pSFV4-T99/45-d1EGFP, pSFV4-d1EGFP-M96, pSFV4-d1EGFP-M37, pSFV4-d1EGFP-M26 and pSFV4-d1EGFP-M21; cells transfected with transcripts from pSP6-SFV4 were used as controls. Cells were collected at 12 h post-transfection, lysed in Laemmli buffer, and subjected to SDS-PAGE in 12% gel; each line corresponds to material from 50,000 cells. Proteins were transferred to nitrocellulose filter, probed by corresponding polyclonal antisera, and visualized by ECL. Sections of the blots probed by anti-SFV-nsP1 antiserum (top), anti-EGFP antiserum (middle) and anti-SFV-capsid antiserum (bottom) are shown. The bands corresponding to detected proteins are indicated with arrows on the left; the names of replication-competent vectors are shown above the blot. SFV4 indicates cells transfected with infectious transcripts from pSP6-SFV4.

To analyse the functionality of constructed vectors, BHK-21 cells were transfected with infectious transcripts prepared from all eight constructs, and the transcripts from pSP6-SFV4 [46] were used as a control. The efficiency of the transfection procedure was reproducibly close to 100% (data not shown). The analysis revealed that, as expected, all vectors expressed nsP1 at comparable levels (Figure 2), which indicated efficient replication in transfected cells. In contrast, the level of d1EGFP expression was different: no d1EGFP expression was detected by immunoblotting (Figure 2) or by fluorescent microscopy in SFV4-T21/5-d1EGFP transfected cells, and only very faint d1EGFP expression was detected for this vector by use of flow cytometry (data not shown). At the same time, d1EGFP expression was detected clearly in cells transfected by other vectors. For terminal vectors, the highest level of d1EGFP expression was detected in SFV4-T99/45-d1EGFP-transfected cells, while the d1EGFP levels for SFV4-T26/20-d1EGFP and SFV4-T37/17-d1EGFP were lower and similar to each other (Figure 2). Thus, in the case of terminal vectors, the d1EGFP expression correlated with length of duplicated SG promoter. In the case of all middle vectors, the d1EGFP expression was derived from identical SG promoters. Nevertheless, its expression levels in transfected cells were not identical: a reverse correlation between d1EGFP expression level and the length of duplicated SG promoter was observed (Figure 2). This tendency is similar to that previously observed in SINV vectors [20], and most likely reflects the consequence of competition between SG promoters placed close to each other.

With the notable exception of SFV4-d1EGFP-M21, all replication-competent vectors with duplicated SG promoters produced capsid protein at a level similar to that for SFV4 (Figure 2). The growth kinetics of these vectors in transfected cell culture were similar to those of SFV4 (data not shown), and the titres of collected stocks reached 2 × 10^8^–5 × 10^8 ^pfu/ml at 12 h post-transfection. In SFV4-d1EGFP-M21-transfected cells, the levels of capsid protein were significantly lower (Figure 2), the release of infectious virions into the growth medium was delayed by 4 h, and the virus titre at 12 h post-transfection was approximately 100-fold lower than that for other vectors. In addition, very few (<1%) of the cells infected at multiplicity of infection (moi) of 1 with collected stock of SFV4-d1EGFP-M21 were d1EGFP-positive. Taken together, these data indicated that both 21/5 and 21/51 SG promoters were not functional (or had extremely low activity) in their native context. Therefore, SFV4-d1EGFP-M21 was able to replicate its RNA but not to produce structural proteins and consequently to form infectious virions. This resulted in huge selection pressure for re-positioning of the expression of the structural region under the control of an active native SG promoter. The plasticity of the alphavirus genomes allowed the required changes to take place very rapidly; the 4-h delay, observed in virion formation for SFV4-d1EGFP-M21, is in agreement with the time required for SFV4 to accumulate compensatory changes [47]. Thus, the capsid protein detected in SFV4-d1EGFP-M21-transfected cells was most likely produced by recombinant genomes that have gained the ability to express structural proteins and have lost the ability to express d1EGFP. The easiest way to achieve this is the deletion of the inserted sequence by copy-choice recombination between native SG promoter sequences and its duplicated copy, which, in the case of SFV4-d1EGFP-M21, shared an identical region of 51 bases. It was confirmed by RT-PCR and sequencing that exact deletion of inserted d1EGFP and duplicated SG promoter had occurred already in SFV4-d1EGFP-M21-transfected cells, and mutants carrying such a deletion became dominant in the collected stock (data not shown). However, it remains possible that other mechanisms such as selection of compensatory mutations may also have contributed to the activation of structural protein expression.

### Construction and viability of SFV4-based replication-competent vectors with inserted IRES elements

The length and positioning of duplicated SG promoters in the genome of the replication-competent vector allows modulation of the level of expression of the protein of interest (Figure 2). In contrast, the time of its expression is strictly determined by the start of SG mRNA synthesis. The IRES of encephalomyocarditis virus (EMCV) has been shown previously to be functional in SINV-infected cells [42], as well as in the context of DNA-RNA layered SINV replicon vectors [30] and SFV VLP vectors [29]. In addition, an IRES element from crucifer-infecting tobamovirus (CR IRES) is also functional in SFV VLP systems [29]. Thus, the use of IRES elements in replication-competent alphavirus vectors represents an attractive option for early and efficient expression of the gene of interest. Nevertheless, with the exception of construction of vertebrate-specific VEEV genomes [32], their use in replication-competent alphavirus vectors has not been described.

**Figure 3:**
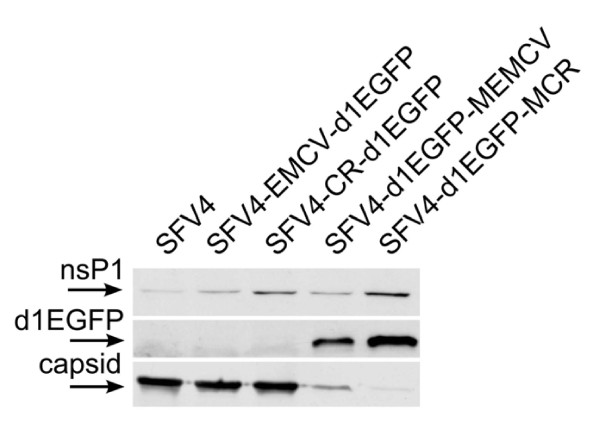
**Protein expression in BHK-21 cells transfected with replication-competent vectors containing inserted IRES elements**. BHK-21 cells were transfected with equal amount of *in vitro *transcripts prepared from pSFV4-EMCV-d1EGFP, pSFV4-CR-d1EGFP, pSFV4-d1EGFP-MEMCV and pSFV4-d1EGFP-MCR, and cells transfected with transcripts from pSP6-SFV4 were used as controls. Analysis was carried out and materials are presented as described in Figure 2.

To test such possibility, we first constructed vectors where EMCV IRES or CR IRES with d1EGFP marker were inserted into the 3'-UTR of SFV4 (Figure 1B). In transfected cells, these vectors, designated SFV4-EMCV-d1EGFP and SFV4-CR-d1EGFP, expressed normal levels of nsP1 (for some unknown reason, SFV4-CR-d1EGFP expressed more nsP1 than did SFV4-EMCV-d1EGFP) and capsid protein (Figure 3). The growth kinetics and the final titres of collected stocks were both similar to those of SFV4 (data not shown). At the same time, no expression of d1EGFP was detectable by any method including fluorescent microscopy, flow cytometry (data not shown) or immunoblotting (Figure 3).

In order to verify if the observed lack of d1EGFP expression was the consequence of positioning IRES elements downstream of structural region of SFV4, the vectors designated as SFV4-d1EGFP-MEMCV and SFV4-d1EGFP-MCR (Figure 1B) were constructed and analysed. In transfected cells, efficient expression of nsP1 and d1EGFP marker was detected (Figure 3). At the same time, the expression of capsid protein (Figure 3), growth kinetics and titres of collected viral stocks were reminiscent of those of SFV4-d1EGFP-M21, except that capsid protein expression, and consequently, the titres of collected stocks (approximately 1×10^4^–4×10^4 ^pfu/ml) were even lower. Thus, the IRES elements were also not functional in the intergenic region of replication-competent SFV4-based vectors. The expression of structural proteins was restored in SFV4-d1EGFP-MEMCV- and SFV4-d1EGFP-MCR-transfected cells by deletion of inserted sequences; however, in these cases, the deletion was less efficient than for SFV4-d1EGFP-M21. The most likely explanation for this is that, unlike SFV4-d1EGFP-M21, SFV4-d1EGFP-MEMCV and SFV4-d1EGFP-MCR do not contain duplicated sequences, which excludes the possibility of deletion by a homologous copy-choice recombination mechanism.

The lack of detectable IRES-derived marker expression in the case of SFV4-d1EGFP-MEMCV and SFV4-d1EGFP-MCR could not be attributed to the position of IRES with respect to the native SG promoter, the length of spacer between these two elements, or to the positioning of the start codon of the downstream ORF with respect to the IRES, since all these parameters were identical to the previously used SFV VLP systems [29]. The only difference was the sequence, placed under the control of IRES elements, which was the structural region of SFV4 instead of bcl2 or hcRed in previously described VLP vectors. There were significant differences between those sequences in terms of length and their secondary structure. Only the secondary structure of the structural region can be modified in replication-competent vectors. Notably, the RNA region, which encodes for the first 31 N-terminal amino acid residues of the capsid protein, forms a strong hairpin structure. Since the hairpin in alphavirus SG mRNA and IRES elements are used by viruses to ensure the translation of their mRNAs in cells in which translation of cellular messages has stopped [41], they may interfere with each other. To test that possibility, the secondary structure of SFV4 capsid hairpin was destabilized by introducing 24 silent mutations into a 93-bp region. These changes resulted in a decrease of predicted minimum free energy for the corresponding fragment of RNA molecule from -40.2 to -11.74 kcal/mol (prediction made by Vienna RNA package RNAfold Webserver software; ), and by analogy with similar manipulation made in the context of the SINV genome [41], should eliminate the functioning of this element in non-typical translation initiation. Destabilization of the capsid hairpin structure greatly increased the intrinsic instability of pSP6-SFV4 plasmid-based constructs, most likely because it enhanced the translation of toxic structural proteins of SFV4 in *Escherichia coli*. To overcome this problem, all corresponding elements were transferred to pCMV-SFV4 [13], and the resulting infectious plasmids were designated pCMV-SFV4-d1EGFP-MEMCV-HRP^- ^and pCMV-SFV4-d1EGFP-MCR-HRP^-^(replicating vectors, released from these plasmids, are shown in Figure 1B as Middle2 vectors). When transfected into BHK-21 cells, both plasmids were capable of initiating replication of the vector and d1EGFP expression (data not shown); however, in contrast to pCMV-SFV4, they produced an extremely low amount of capsid protein and infectious virus. Therefore, it was concluded that the destabilization of capsid hairpin structure did not result in activation of IRES function.

### Multiplication and RNA synthesis of the replication-competent SFV vectors

The nine replication-competent vectors constructed in this study produced infectious progeny, which, upon re-infection of BHK-21 cells, reproduced the properties of the original *in vitro *transcripts. Their ability to replicate in BHK-21 cells was analysed by two different methods.

**Figure 4:**
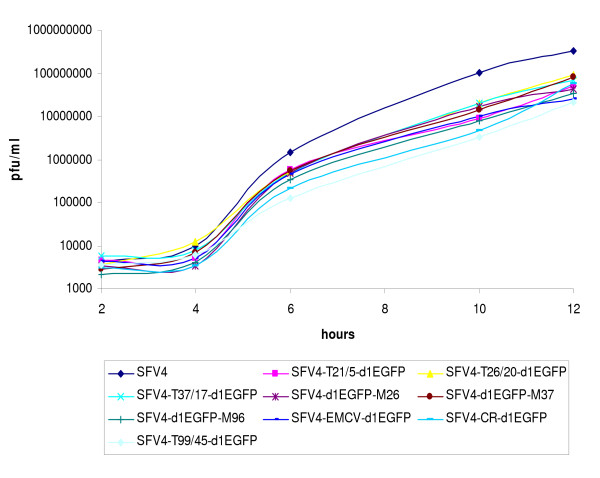
**Growth curves of replication-competent SFV4 vectors**. BHK-21 cells were infected with P1 stocks of SFV4-T21/5-d1EGFP, SFV4-T26/20-d1EGFP, SFV4-T37/17-d1EGFP, SFV4-T99/45-d1EGFP, SFV4-d1EGFP-M26, pSFV4-d1EGFP-M37, pSFV4-d1EGFP-M96, SFV4-EMCV-d1EGFP, SFV4-CR-d1EGFP or SFV4 at an moi of 0.1. Aliquots of culture medium were collected at 2, 4, 6, 10 and 12 h post-infection, and the amount of virus in aliquots was analyzed by plaque titration. The colour and symbols used to present the growth curve of each vector are shown below the figure. Results from one reproducible experiment are presented for clarity.

First, growth curves for these vectors were built and analysed (Figure 4). The growth curves of all replication-competent vectors were similar to each other, and their titres observed 12 h post-infection were somewhat reduced in comparison to those of parental SFV4. These properties are similar to those of other SFV vectors that contain an EGFP insertion [18], and may represent a consequence of increased genome sizes. In general, vectors that contained the largest duplicated SG promoters or IRES elements exhibited slightly slower growth and 2–3-fold lower final titres than vectors with smaller duplicated SG promoters. At the same time, no clear differences between the terminal and middle vectors that contained duplicated SG promoters of the same type (-26, -37 or the largest SG promoter) were observed (Figure 4).

**Figure 5:**
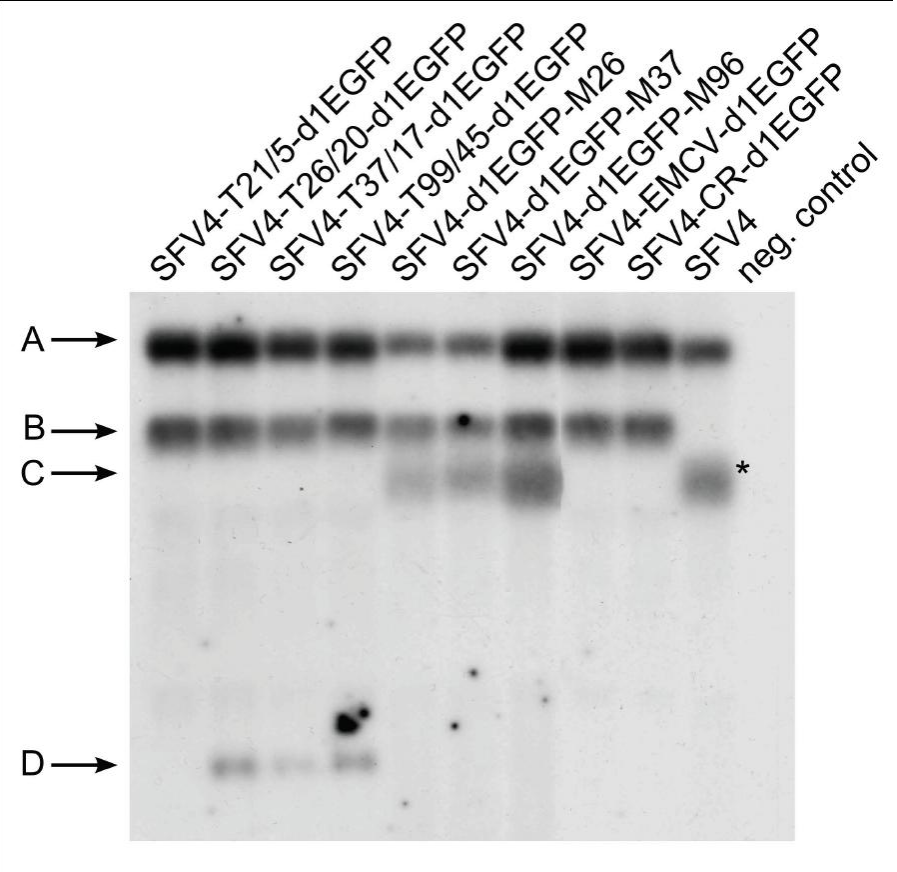
**Accumulation of viral RNA in BHK-21 cells infected with replication-competent vectors**. BHK-21 cells were infected with P1 stocks of SFV4-T21/5-d1EGFP, SFV4-T26/20-d1EGFP, SFV4-T37/17-d1EGFP, SFV4-T99/45-d1EGFP, SFV4-d1EGFP-M26, pSFV4-d1EGFP-M37, pSFV4-d1EGFP-M96, SFV4-EMCV-d1EGFP, SFV4-CR-d1EGFP or SFV4 at an moi of 5. Control cells were mock-infected. Cells were harvested at 12 h post-infection. Total RNA was purified by TRIzol reagent and subjected to agarose electrophoresis and Northern blotting (10 μg total RNA per line). Letters and arrows on the left indicate positions of genomic RNA of vector or virus (A); SG mRNA expressed from native SG promoter (B, except for SFV4 where this mRNA is indicated with an asterisk); SG mRNA expressed from duplicated SG-promoter in middle (C) and terminal (D) vectors. The names of replication-competent vectors are shown above the blot. SFV4 indicates control cells infected with parental SFV4; neg. control indicates RNA from mock-infected cells.

Second, the accumulation of viral RNAs in infected BHK-21 cells was analysed by Northern blotting. This analysis revealed that all replication-competent vectors produced genomic RNAs and SG mRNAs with the expected size. No major additional RNA species were detected for these vectors, which indicated that the passage 1 (P1) stocks used for this experiment were homogeneous (Figure 5). The amount of genomic RNA produced by the constructed vectors was comparable to that of parental SFV4, except that, for SFV4-d1EGFP-M37 and SFV4-d1EGFP-M26, some reduction in the amount genomic RNA (as well as SG mRNAs) was detected. As expected, vectors that contained a duplicated SG promoter produced two SG mRNAs (Figure 5). The exception to this was SFV4-T21/5-d1EGFP, for which a second SG mRNA was not detected, even with prolonged exposition of the blot (data not shown). Thus, it was again confirmed that, in contrast to the 19/5 SG-promoter of SINV, the 21/5 SG-promoter of SFV4 was extremely weak. Expectedly, SFV4-EMCV-d1EGFP and SFV4-CR-d1EGFP produced only a single SG mRNA and, judged by the size, the IRES-d1EGFP insertion was maintained.

### Genetic stability of replication-competent SFV4-based vectors

In order to be useful, the replication-competent vector must maintain its ability to express the gene of interest over several generations. Accumulation of point mutations in inserted sequences caused by the high error rate of RNA polymerases depends on the length of the inserted sequence and not on the design of the vector. At the same time, this process does not result in an increase in genomes with significant growth advantages over the original replication-competent vector. Therefore deletions of the inserted sequences, which results in at least three important advantages for the mutated genome, are the main factor that reduces genetic stability of the vector. Namely: (i) smaller sizes facilitate packaging of the genome; (ii) elimination of inserted expression units restores the wild-type regulation and coordination of viral RNA and protein synthesis; and (iii) shorter genomes can replicate faster. Thus, wild-type-like virus that can produce up to 10-fold more infectious progeny (Figure 4) is capable of superseding the original replication-competent vector. The efficiency of this process depends on the conditions of virus propagation. In the case of high moi, the competition takes place only inside the infected cells, and under these conditions, the high molar excess of original vector genomes reduces the growth advantage of the deletion mutants. In contrast, low moi involves spreading of the infection in cell culture and therefore strongly favours wild-type-like sequences.

These considerations were experimentally verified by the use of a set from six replication-competent vectors. In the first experiment, SFV4-T26/20-d1EGFP, SFV4-T37/17-d1EGFP and SFV4-T99/45-EGFP were propagated five times at an moi of 10 in BHK-21 cells, and the percentage of d1EGFP-positive cells in each passage was measured by flow cytometry. All cells infected with corresponding P5 stocks were found to be d1EGFP-positive (e.g., they were infected by at least one particle capable of initiating d1EGFP expression) (data not shown). In the second experiment, in which the stability analysis was carried out at low moi [18,19,23], clear differences in stability of replication-competent vectors were revealed (Table 1). Since the d1EGFP fluorescence in infected cells was transient and relatively weak, genome stability of the replication-competent vectors was also analysed by RT-PCR. Results of RT-PCR analysis were highly coherent with those presented in Table 1 and revealed that the decrease in the percentage of d1EGFP-positive plaques correlated with the percentage of genomes with deletion of the inserted marker (data not shown). The main conclusion from this experiment is that all terminal vectors of SFV4 were more stable than their middle vector counterparts. The differences could not be attributed to differences in growth properties (Figure 4); instead, they may have originated from the different outcome of homologous copy-choice recombination. In the case of middle vectors, the homologous recombination between native and duplicated SG promoters resulted in re-creation of the wild-type SFV4 genome. In the case of terminal vectors, the same process resulted in deletion of the structural region. It can be seen that, at least for terminal vectors, the vectors with shorter duplicated SG promoters were more stable than those that contained the longest duplicated SG promoter. The most likely reason for this was the slower growth rate of the latter (Figure 4), which increased growth advantage of deletion mutants over the original vector genomes.

**Table 1:** Comparison of genetic stability of different replication-competent SFV4-based vectors in BHK-21 cells

	**P1**	**P2**	**P3**	**P4**	**P5**
**SFV4-T37/17-d1EGFP**	100%	100%	100%	80%	70%

**SFV4-T26/20-d1EGFP**	100%	96%	85%	59%	41%

**SFV4-T99/45-d1EGFP**	96%	75%	65%	41%	15%

**SFV4-d1EGFP-M26**	100%	50%	11%	0%	0%

**SFV4-d1EGFP-M37**	96%	64%	38%	3%	0%

**SFV4-d1EGFP-M96**	96%	50%	19%	11%	0%

The d1EGFP expression was transient and relatively weak. Therefore, it is possible that some d1EGFP positive plaques were counted as d1EGFP negative and the stability of constructs was somewhat under-estimated.

### *In vitro *ligation/transcription for construction of replication-competent SFV vectors and libraries, based on these vectors

The plasmid for infectious cDNA of SFV4, pSP6-SFV4 is unstable in *E. coli *cells. This property is maintained or even enhanced for plasmids that contain cDNAs of replication-competent vectors. To overcome this problem, we have previously developed a stable pCMV-SFV4 plasmid, which can be used for construction of SFV replication-competent vectors. However, its infectivity is lower than that for infectious transcripts [13], especially in cells in which the CMV promoter is weak. Thus, pCMV-SFV4 represents a useful alternative to pSP6-SFV4, but has limitations of its own.

**Figure 6:**
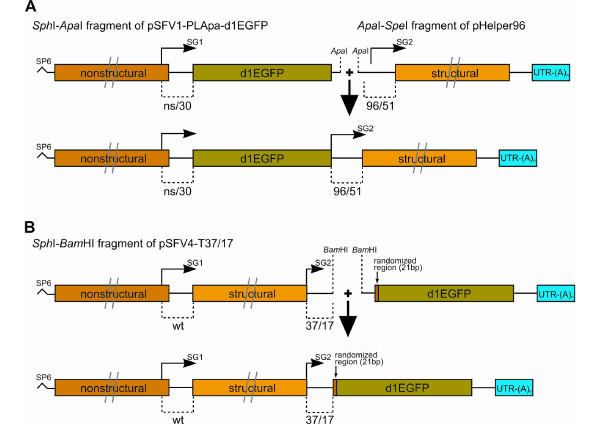
**Schematic presentation of the methods for replication-competent vector genome and library construction**. (A) Construction of replication-competent vector from restriction fragments of pSFV-PLApa-d1EGFP (*Sph*I to *Apa*I) and pHelper96 (*Apa*I to *Spe*I). SP6 corresponds to the promoter for SP6 RNA polymerase; bold arrow indicates the ligation process. (B) Construction of library from *Sph*I/*Bam*HI restriction fragment of pSFV4-T37/17 and *Bam*HI-treated product of PCR-based mutagenesis, which contained 21-bp randomized fragment, coding sequence of d1EGFP and SFV4 3'-UTR with poly(A). SP6 corresponds to the promoter for SP6 RNA polymerase and bold arrow indicates the ligation process. Symbols have the same meaning as in Figure 1B. Only the ligation products corresponding to the cDNAs of replication-competent vector (A) or replication-competent vector-based library (B) are shown.

In contrast to pSP6-SFV4, pSFV1 and pHelper1 are stable in *E. coli *and this property is preserved in the majority of constructs based on these plasmids. Thus, if the elements required for foreign gene expression are included in one of derivatives of these plasmids, it may be possible to rejoin it with another part of the SFV4 genome and use the resulting molecule directly for *in vitro *transcription. One possibility to achieve this is to use PCR. Unfortunately, PCR always lead to generation of a pool of heterogeneous sequences that contain random PCR mutations, which can have unpredictable effects on the properties of the replication-competent vectors. Therefore, instead of using PCR, we designed vectors in which a unique *Apa*I restriction site was located downstream of d1EGFP in pSFV1-PLApa-d1EGFP -and upstream of duplicated SG promoter in pHelper96, pHelper37, pHelper26 and pHelper21. The fragment that contained the SP6 promoter, the ns region of SFV4 and d1EGFP was cut out from pSFV1-PLApa-d1EGFP by *Sph*I/*Apa*I restriction and ligated to the *Apa*I/*Spe*I fragment from pHelper96 that contained a duplicated SG promoter, structural region, and a 3'-UTR with the poly(A) of SFV4 (Figure 6A). The products of the ligation reaction were transcribed *in vitro *and, since only the transcript corresponding to the genome of the replication-competent vector contained all the elements needed for initiation of replication, the mixture was directly used to transfect BHK-21 cells. Analysis of transfected cells by fluorescent microscopy revealed that d1EGFP expression and replication were initiated in a large number of cells. By 24 h post-transfection, all cells showed the symptoms of infection and the collected virus stock had a titre and properties similar to the stock obtained from cells transfected by transcripts from pSFV4-d1EGFP-M96. Thus, the *in vitro *ligation/transcription procedure can be used for construction of genomes of replication-competent SFV vectors.

pSP6-SFV4-based libraries, propagated as a pool in *E. coli*, are invariably and rapidly overgrown by bacteria containing defective plasmids. Therefore, another possible application of the developed *in vitro *ligation/transcription procedure is its usage for constructing SFV4 replication-competent vector-based libraries. The first option for doing this is to convert VLP-replicon libraries, generated by the use of pSFV1-PLApa or similar vectors, to replication-competent vector libraries by the use of the above approach. This will, however, result in replication-competent vectors that carry inserted sequences in the middle position that, according to our data (Table 1), are relatively unstable. Nevertheless, the approach can be used if the vector instability is not a serious problem or it is overcome by changes in vector design. The second option is to generate libraries using terminal vectors. To demonstrate the feasibility of this approach, 1 μg of the *Sph*I/*Bam*H1 restriction fragment from pSFV4-T37/17 vector was ligated with a *Bam*H1-digested PCR fragment that contained a 21-bp randomized region, d1EGFP encoding sequence, and a 3'-UTR and poly(A) tail of SFV4 (Figure 6B). The ligation product was transcribed *in vitro *and used for transfection of BHK-21 cells. The number of initially transfected cells (corresponding to the number of replication-competent vectors with different insertions in the library) was measured by an infectious centre assay. After the optimisation of DNA purification, ligation and transcription procedures, libraries that contained 2×10^5^–5×10^5 ^different replication-competent vector genomes were obtained per single *in vitro *ligation/transcription procedure. These libraries were subsequently amplified to high titre, propagated up to five times at an moi of 0.1, and analysed again for their ability to express d1EGFP and the presence of randomised sequences. The results of this analysis confirmed that the inserted sequences were stable in the pSFV4-T37/17-based library. Thus, the *in vitro *ligation/transcription procedure can be used for construction of large SFV replication-competent-vector-based expression libraries that can be subsequently propagated and subjected to various analyses.

## Discussion

In contrast to the minimal 19/5 SG-promoters of several alphaviruses, the corresponding element of SFV4 has a G residue at the -1 position of its sequence, and it is not functional in the context of a replication-competent vector of SINV. It has been hypothesized that, under native conditions, this change may be compensated by other changes in the virus genome [24]. Indeed, it has been shown that changes in *cis-*elements of alphaviruses can be compensated by changes in replicase proteins and *vice versa *[48,49]. The data presented above did not, however, support this hypothesis, since the 21/5 SG promoter of SFV4 had no detectable activity in the context of SFV4-derived replication-competent vectors (Figure 5). Furthermore, the extreme instability of the SFV4-d1EGFP-M21 suggests that the even longer, 21/51 SG promoter of SFV4 was much less active than the minimal SG promoter of SINV, which has been used in SINV vectors of analogous design. Although the specific impact of the sequences located downstream of the +5 position on the activity of SFV4 SG promoter was not studied in detail, it is clear that if these elements contributed to the activity of the SG-promoter, their effect was rather small. Again, this differs from the data obtained for SINV, in which the region located between positions +6 and +14 significantly contributes to the activity of SG promoters [27]. Thus, the minimal SG promoter of SFV4 must contain more than 21 upstream residues. Three different regions upstream of the +21 position were identified (Figure 1A): the -30/-35 region, which is conserved in SG promoters of alphaviruses [27], and two non-conserved regions, located downstream (-29/-22) and upstream (ns/-36) of the conserved element. The analysis of SG mRNA synthesis revealed that T26/20, T37/17 and T99/45 SG promoters, as well as 26/51, 37/51 and 96/51 SG promoters, were active and there were no major differences in their activities (Figure 5). Thus, the effect of the -1 G residue in the SG promoter of SFV4 was compensated by the unique sequences located in the -22 to -26 region.

The levels of SG mRNA produced by duplicated SG promoters in SFV4-T99/45-d1EGFP- and SFV4-T26/20-d1EGFP-infected cells were similar to each other (Figure 5), but d1EGFP expression was significantly higher in cells transfected with the former construct (Figure 2). The most likely explanation for this discrepancy is that the region located between +21 and +45 had little or no effect on the activity of SG promoter, but it did affect positively the translation of corresponding SG mRNA, as has been described for SINV SG promoter and SG mRNA [37,42].

The region between -96 and -27 had a small effect on transcription activation (Figure 5). Since the detailed analysis of SG mRNA synthesis was not in the scope of this study, the exact significance of this region remains to be established. Similarly, the precise mapping of the truly minimal SG promoter of SFV4, estimation of its activity relative to the full-size SG promoter, and identification of the exact positions of critical residues that compensate for the effect of the -1 G residue represent topics for further studies.

The analysis of genetic stability of the constructed SFV4-based replication-competent vectors (Table 1) revealed that, in contrast to the related Chikungunya-virus-based vectors [23], the terminal vectors of SFV4 were invariably more stable than their middle-type counterparts. It is possible that the stability of replication-competent alphavirus vectors may depend on their construction, for example, from the exact position of duplicated elements and/or from the genetic background of the vector itself. Our analysis also indicated that the loss of the ability to express a functional gene of interest correlates with the loss of the gene (or full expression unit), and is most likely caused by gradual superseding of the original replication-competent vector by its faster-growing deletion variants. Thus, the stability of replication-competent vectors depends on the frequency of formation of replication-competent genomes with deletions and on their growth advantage over the original vector. Both these factors may contribute to the lower genetic stability of the middle vectors because the homologous copy-choice recombination between SG promoters results in the wild-type virus genome, which has the biggest growth advantage over the replication-competent vector. In the case of terminal vectors, only non-homologous recombination events, which do not result in reversion to exact wild-type genome, can take place. However, other factors such as slightly larger sizes of duplications, and stronger interference between native and inserted elements cannot be excluded as reasons behind lower stability of middle vectors.

The fact that the appearance of deletion variants of the original vector and their subsequent increase in population of replicating genomes are the most important factors for vector instability suggests that conditions for propagation of replication-competent vectors must be carefully selected. In contrast to the common practice with propagation of viral stocks, when low moi is used to avoid an increase in defective interfering (DI) genomes, the replication-competent vectors should be propagated under high moi conditions. The deletion variants of replication-competent vectors are, in contrast to DI genomes, fully competent for replication and particle production, and have an increased growth advantage when the propagation involves spreading in cell culture. Alternatively, the size of sample used for vector propagation, should be reduced. Our data on the stability of recombinant stocks (Table 1) indicate that, in general, P1 stock contains less than 1% defective vectors. Northern blot analysis revealed the same (Figure 5), which indicates that the initial appearance of deletion mutants is a relatively rare event. Thus, if the size of the sample used for propagation of the replication-competent vector stock is kept sufficiently small that it does not already contain deletion versions, then the probability of their formation remains low, and they will less likely become dominant in the population.

The typical error rate for replicases of RNA viruses is estimated to be as high as 1 error per 10,000 bases. Taking into account that RNA replication consists of negative-strand synthesis followed by positive-strand synthesis, and that, with a low moi, this cycle is repeated more than once, one could expect four or more changes per 10,000 bases during replication in a single cell. Thus, under the conditions used for vector propagation (moi 0.1), the ~1000-bp SG promoter d1EGFP insert would likely acquire more than one point mutation during five passages of the stock. Therefore, at least in part, the loss of functional d1EGFP expression in P4 and P5 stocks may be caused by accidental point mutations formed by RNA replicase. The speed of functional inactivation of any specific protein of interest depends on its size and sensitivity to miss-sense mutations, but it is doubtful that any expression vector based on RNA viruses (with the exception of coronaviruses, which have a proofreading-like function [50]) can be propagated more than 5–10 times under low moi conditions.

In a number of studies, dedicated to the construction of replication-competent vectors, the superior stability of novel vectors has been claimed. However, the stability of different replication-competent alphavirus vectors can only be compared if they have been propagated and analysed under similar conditions. Among vectors that have been propagated and analysed in the same manner as in the present study, the most stable ones are CHIKV-LR 5'GFP [23] and SFV4(3H)-eGFP [19]; both of these produce nearly 95% EGFP-positive plaques after five passages in BHK-21 cells. Thus, SFV4-T37/17-d1EGFP, which was the most stable replication-competent vector constructed in the present study (Table 1), also represents a vector with excellent genetic stability.

The use of IRES elements was considered as an alternative for SG promoter insertion, since it avoids duplication of viral sequences and allows early protein expression directly from genomic RNA of vectors. IRES elements and the d1EGFP sequence placed under their control were well tolerated and maintained in terminal vectors (Figure 5), but in all cases, the IRES elements themselves were non-functional, regardless of their origin (EMCV IRES or CR IRES) and insertion site. In contrast, EMCV IRES inserted into the VEEV genome is functional and can be used to direct synthesis of the structural proteins of this virus [32]. IRES-containing VEEV also has an interesting functional defect: despite effective replication and structural protein expression, it produces very few infectious virions. This defect is compensated by mutations in nsP2 but the mechanism of compensation is not fully understood [32]. Currently, it is unclear if the selection for compensatory mutations can be used to activate the IRES-derived expression in the case of replication-competent SFV4-based vectors. An alternative approach, the removal of the hairpin structure of SG mRNA, which can functionally or structurally interfere with the activity of IRES elements, failed to restore IRES activity. Thus, in contrast to the alphavirus-derived VLP or DNA-RNA layered vectors, IRES elements did not function in SFV4-derived replication-competent vectors. The mechanisms responsible for this phenomenon, as well as those responsible for differences between SFV4 and VEEV-based systems remain unknown.

The infectivity of naked genomic RNA simplifies the construction of highly representative expression libraries based on an alphavirus VLP system. However, such libraries have several problems. First, the suicidal nature of these VLP-system vectors does not allow propagation of the library. To some extent, this problem can be overcome by the use of packaging cell lines [51] or three-component vector systems described for SINV vectors [52]. Second, in general, these systems do not produce infectious progeny in cells used for functional analysis of expressed proteins. Consequently, the vector's genetic material must be extracted from selected cells and analysed. If functional analysis requires multiple cycles of selection, it becomes time consuming and complicated. Therefore replication-competent vectors, which can be propagated and produce infectious progeny in any infected cell, greatly simplify the analysis and selection of viruses during multiple cycles of analysis. On the other hand, specific problems such as stability and the packaging limit for such a vector are typical for replication-competent vector-based libraries. Thus, the approach that allows easy conversion of VLP-based expression libraries to replication-competent vector libraries (Figure 6A) and combines benefits of both systems, may be useful. The same approach can also be used for conversion of any single VLP-type vector into a replication-competent vector.

The single-step *in vitro *ligation/transcription procedure offers an alternative for constructing VLP-based libraries and their subsequent conversion to replication-competent vector libraries. In the case of middle vectors, it is required that three fragments, which represent the replicase, insert (library) and structural part, are joined in one reaction. The efficiency of this process was much lower (data not shown) than for terminal vectors, for which only two fragments had to be joined (Figure 6B). In the latter case, the second fragment should, however, contain sequences required for alphavirus replication. In the present study, this was achieved by the use of plasmid vectors that contained a 3'-UTR of SFV4 followed by a poly(A) tract. This may not have been necessary since the essential 3'-elements required for replication of the alphavirus genome consist only of 19 conserved 3'-residues and a poly(A) tract that contains 12 A residues [53]. Such 31-base sequences can be included in primers used for library generation. pSFV4-T26/20, pSFV4-T37/17 or pSFV4-T99/45 can be used as vectors for the library. Even the use of pSFV4-T21/5 can be envisaged for cases where very low expression levels of inserted genes are required. The *in vitro *ligation/transcription procedure was efficient and allowed us to obtain highly representative libraries that contained hundreds of thousands of different viral clones (or more, if more material was used for ligation/transcription, or if the procedure of joining two fragments were further optimised). It was also found that expression cassettes of other types of SFV4-based vectors such as SFV4(3H)-eGFP or SFV4-steGFP [19] could be combined with any terminal vector that allowed expression of two foreign proteins (data not shown). For example, the first cassette can be used for expressing a marker, which allows identification/purification of infected cells (or for expressing a reporter to provide an internal standard), while the duplicated SG promoter of the terminal vector may be used for expression of the library. The downside of this approach is the reduced capacity of such vectors (decreased by the size of the first expression cassette), and the possibly reduced genetic stability caused by the large size of the genome.

## Conclusion

The data obtained in this study allow us to conclude that the minimal functional SG promoter of SFV4 is organised differently from that of SINV and other alphaviruses. The presence of a G residue at the -1 position of the SG promoter of SFV4 is not compensated by changes in virus-encoded proteins, as previously hypothesised. Instead, the minimal SG promoter of SFV4 is longer than those of other alphaviruses and includes residues located in the -22 to -26 region. The presence of residues located upstream of position -27 and possibly, residues located downstream of position +5, has a smaller effect on the activity of the SG promoter of SFV4 than that of SINV [27].

It was found that when constructed vectors were unable to express viral structural proteins at the level required for virion formation, genetic rearrangements that led to restoration of such expression had taken place in initially transfected cells. In total, five vectors analysed in the present study suffered from this problem. In all of these vectors, extreme instability of their genomes was observed: the inserted sequences were lost and expression of structural proteins was switched back to the native SG promoter. In contrast, all vectors capable of structural protein expression formed essentially homogeneous stocks in initially transfected cells.

By duplicating the SG promoter sequence, six replication-competent SFV4-based vectors, which were capable of virion formation and expression of inserted markers, were obtained. The genetic stability of these vectors, as well as RNA synthesis, protein expression, virus rescue and growth kinetics, was analysed and compared with data obtained for eight defective vectors. These data allow us to conclude that the loss of ability to express functional markers by replication-competent vectors is mainly caused by the loss of inserted sequences. The loss of activity of inserted proteins, caused by accumulation of point mutations that result from the lack of proofreading function of RNA replicase, may be important only for the most stable replication-competent vectors.

The growth advantage of genomes with deletions over the original genomes of the replication-competent vectors is the main driving force for the elimination of inserted sequences. When the loss of inserted sequences is attributed to the homologous copy-choice recombination between native and inserted sequences, reduced genetic stability of the vector is observed. This may be a consequence of higher efficiency of homologous recombination compared to the mechanisms of deletion by non-homologous recombination, or it may result from the fact that homologous recombination reverts the vector to the wild-type SFV4 (which has the biggest growth advantages over the vectors with inserts). These tendencies should be taken into account for design of stable and efficient replication-competent vectors of SFV4 and for selection of conditions for their propagation.

Comparison of reported properties of replication-competent vectors of different designs and origin reveals a contradictory picture. In part, these contradictions may be attributed to the different methods used for the analysis of constructed vectors; however, it is likely that the number of discrepancies reflects unique properties of individual viruses and their isolates (exemplified by the unique structure of the SG promoter of SFV4). Therefore designs that work efficiently for one group of alphavirus-based vectors may not necessarily do so in others. Furthermore, the designs used for the replicon (VLP or DNA-RNA layered) vectors may not work in the same way for replication-competent vectors (exemplified by the different activities of IRES elements).

Replication-competent vectors, including those of SFV4, represent tools for cloning and functional analysis of expression libraries. In cases where such libraries cannot be created by obtaining a plasmid library and subsequent rescue of replication-competent vectors, the method of *in vitro *ligation/transcription developed in the present study can be used. The method can also be used for conversion of VLP-type replicon-vector libraries to replication-competent vector libraries, and may use the benefits of both types of vectors.

## Methods

### Cells and medium

BHK-21 cells were grown in Glasgow's Minimal Essential Medium (GMEM) that contained 5% foetal calf serum (FCS), 0.3% tryptose phosphate broth, 0.1 U/mL penicillin and 0.1 μg/mL streptomycin in an incubator at 37°C and 5% CO_2_.

### Viral sequences and clones

For construction of replication-competent vectors of SFV4, the following plasmids that contained cDNA of SFV4 were used: pSP6-SFV4 [46], pSFV1, pHelper1 [3] and pCMV-SFV4 [13]. The sequences of EMCV IRES (pIRES2-EGFP; BD Clontech) and the 148-bp CR IRES [54] elements, and their positioning with respect to the initiation codon were identical to the previously described SFV VLP replicon vectors [29].

### Cloning, PCR and *in vitro *mutagenesis

Standard cloning procedures were used for construction of modified replicon-, helper- and replication-competent vector cDNAs. The oligonucleotide primers were obtained from Sigma-Proligo. PCR-based mutagenesis reaction and PCR amplifications were performed by use of Phusion High-Fidelity DNA polymerase (Finnzymes). All DNA sequences, resulting from PCR reactions, *in vitro *mutagenesis or insertions of oligonucleotide duplexes, were verified by sequencing. Sequences of all clones are available from the authors upon request.

### Construction of SFV4 terminal vectors with inserted d1EGFP marker

To construct replication-competent SFV4 vectors that contained a duplicated SG promoter inserted in a 3'-UTR of SFV4, a polylinker with the sequence GGGCCCGGGGGATCC was inserted in pSP6-SFV4, immediately downstream of the terminator codon of the E1 gene. The resulting plasmid was designated pSP6-SFV4-PL. The full-length (-99/+45) SG promoter was PCR-amplified using primers that contained *Apa*I (upstream) and *Bam*H1 (downstream) recognition sites at the 5' ends, and the sequences of minimal (-21/+5) and medium-sized (-26/+20 and -37/+17) SG promoters were constructed from oligonucleotide duplexes. Duplicated SG-promoter sequences were inserted into the *Apa*I/*Bam*HI-digested pSP6-SFV4-PL to obtain plasmids designated pSFV4-T21/5, pSFV4-T26/20, pSFV4-T37/17 and pSFV-T99/45. The ORF of d1EGFP (BD-Clontech) was fused to the 3'-UTR of SFV4 (including poly(A)_48 _sequence), PCR-amplified using primers 5'-CGGGATCCGAT**ATG**GTGAGCAAGGGCGAGGAGCTGTT-3' (start codon of d1EGFP in bold) and 5'-CGACTAGT(T)_48_GGAAATATT-3', and cloned into *Bam*H1/*Spe*I-digested pSFV4-T21/5, pSFV4-T26/20, pSFV4-T37/17 and pSFV4-T99/45. The resulting plasmids were designated pSFV4-T21/5-d1EGFP, pSFV4-T26/20-d1EGFP, pSFV4-T37/17-d1EGFP and pSFV4-T99/45-d1EGFP.

To construct vectors that contained IRES elements downstream of the region that encodes for structural proteins of SFV4, the EMCV-IRES and the CR-IRES were first fused with the coding sequence of d1EGFP. The obtained sequences were PCR-amplified using primers that contained *Apa*I (upstream) and *Bam*HI (downstream) recognition sites at the 5' ends, and inserted into the *Apa*I/*Bam*HI-digested pSFV4-PL to obtain plasmids designated pSFV4-EMCV-d1EGFP and pSFV4-CR-d1EGFP.

### Construction of SFV4 middle vectors with inserted d1EGFP marker

To construct replication-competent SFV4 vectors that contained duplicated SG promoters inserted in intergenic regions, a polylinker in pSFV1 was replaced by sequences that contained recognition sites for (5' to 3') *Bam*HI, *Nru*I, *Cla*I and *Apa*I, which resulted in plasmid pSFV1-PLApa. To obtain plasmid pSFV1-PLApa-d1EGFP, the sequence that codes for d1EGFP was cloned into pSFV1-PLApa using *Bam*H1 and *Nru*I sites of the polylinker. pHelper1 was used as a template for PCR-based mutagenesis to introduce the recognition site of *Apa*I at 96, 37, 26 or 21 bases upstream of the position corresponding to the 5' end of SG mRNA. This resulted in plasmids that were designated pHelper96, pHelper37, pHelper26 and pHelper21. The cDNA clones of replication-competent vectors with d1EGFP markers were obtained by cloning *Apa*I/*Spe*I restriction fragments from pHelper96, pHelper37, pHelper26 and pHelper21 to pSFV1-PLApa-d1EGFP vector digested with the same enzymes. The resulting clones were designated pSFV4-d1EGFP-M96, pSFV4-d1EGFP-M37, pSFV4-d1EGFP-M26 and pSFV4-d1EGFP-M21.

To construct replication-competent SFV4 vectors that contained IRES elements, a polylinker with the sequence GGGCCCATGCATATACAT**ATG **(which contained recognition sites for *Apa*I, *Nsi*I and *Nde*I, nucleotides in bold correspond to the start codon of SFV4 structural polyprotein) was inserted in pHelper1, and the resulting plasmid was designated pHelperPL. The PCR-amplified fragments of the EMCV IRES and the CR IRES were cloned into *Nsi*I- and *Nde*I-digested pHelperPL, and the resulting plasmids were designated pHelperEMCV and pHelperCR. The cDNA clones of replication-competent vectors with d1EGFP markers were obtained by cloning *Apa*I/*Spe*I restriction fragments from pHelperEMCV and pHelperCR to pSFV1-PLApa-d1EGFP vector digested with the same enzymes. The resulting clones were designated pSFV4-d1EGFP-MEMCV and pSFV4-d1EGFP-MCR.

To destabilize the hairpin structure at the RNA region that encoded for the 31 N-terminal amino acid residues of SFV4 capsid protein, 24 silent mutations were introduced into the corresponding region of pHelperPL, and the resulting plasmid was designated pHelperPL-HRP^-^. The PCR-amplified fragments of EMCV IRES and CR IRES were cloned into pHelperPL-HRP^- ^as described above, and the resulting plasmids were designated pHelperEMCV-HRP^- ^and pHelperCR-HRP^-^. Construction of plasmids that contained cDNAs of the corresponding replication-competent SFV4 vectors using *Apa*I/*Spe*I restriction fragments from pHelperEMCV-HRP^- ^and pHelperCR-HRP^- ^and pSFV1-PLApa-d1EGFP vector was not successful, because of the increased instability of the resulting plasmids in *E. coli*. To overcome this obstacle, all the essential elements of these replication-competent vectors, including the modified polylinker with an inserted d1EGFP marker, IRES element and start region for the structural region with the mutated hairpin element were transferred to pCMV-SFV4 vector [13], and the resulting infectious plasmids were designated pCMV-SFV4-d1EGFP-MEMCV-HRP^- ^and pCMV-SFV4-d1EGFP-MCR-HRP^-^.

### Generation of PCR fragments with randomised sequences

The template plasmid used for random libraries generation contained the coding sequence of d1EGFP, followed by the 3'-UTR of SFV4 and poly(A) of 48 A residues. The random library was created by PCR reaction using an upstream (sense) primer with the sequence 5'-CGGGATCC(N)_21_GAT**ATG**GTGAGCAAGGGCGAGGAGCTGTT-3' (N is a random nucleotide, the start codon for d1EGFP is in bold) and a downstream (antisense) primer with the sequence 5'-CGACTAGT(T)_48_GGAAATATT-3'. PCR reaction was carried out by use of Phusion High Fidelity DNA polymerase (Finnzymes). The 5'-region of the obtained product was sequenced to eliminate the possibility of library shifts.

### *In vitro *ligation

The procedure for construction of replication-competent SFV4 vectors or replication-competent SFV4 vector-based libraries is illustrated by the following examples. The DNA fragment obtained by *Sph*I and *Apa*I digestion of pSFV1-PLApa-d1EGFP (containing promoter for SP6 RNA polymerase, 5'-UTR and ns part of the SFV4 genome, native SG promoter, and cloned d1EGFP marker) was ligated with a DNA fragment obtained by *Apa*I and *Spe*I digestion of pHelper96 (containing duplicated 96/51 SG promoter, structural region of SFV4 genome, and 3'-UTR with poly(A) sequence). For library construction, the DNA fragment obtained by *Sph*I and *Bam*HI digestion of pSFV4-T37/17 (containing the promoter for SP6 RNA polymerase, full-lenght cDNA of the SFV4 genome except for the 3'-UTR and duplicated 37/17 SG promoter) was ligated with a fragment obtained by *Bam*HI digestion of an *in vitro *mutagenesis product (containing a randomised region followed by the coding sequence of d1EGFP, 3'-UTR of SFV4 and poly(A)_48 _sequence). The following conditions of ligation were used: 1 μg of DNA fragment that contained cDNA for the ns region of SFV4, was ligated with 10-fold molar excess of DNA fragment that contained cDNA for the 3' regions of SFV4. Ligation was carried using T4 DNA ligase (Roche) in the presence of PEG at 12°C for 24 h. The ligation products were purified using the PCR Purification Kit (Genomed GmbH) and used as a template for *in vitro *transcription.

### *In vitro *transcription and transfection of cells

Plasmids that contained the cDNAs of SFV4-based replication-competent vectors were linearised by *Spe*I digestion. Alternatively, the products of *in vitro *ligation were used as a template for transcription. Infectious transcripts were synthesized *in vitro *by SP6 RNA polymerase and used for cell transfection by electroporation as described previously [55]. For pCMV-SFV4 and replication-competent vectors, based on this plasmid, we used the transfection procedure described previously [13]. Primary stocks of replication-competent SFV4 vectors or expression libraries were collected from transfected BHK-21 cell cultures at 12 h post-transfection.

### Virological methods

Titres of collected stocks were determined by standard plaque assay. The growth curves of replication-competent SFV4 vectors were analyzed as previously described [18]. For infectious centre assay, we used *in-vitro*-synthesized RNA, obtained by ligation/transcription reaction, for electroporation of 8×10^6 ^BHK-21 cells. Ten-fold dilutions of electroporated cells were seeded in six-well tissue culture plates that contained 1.5×10^6 ^BHK-21 cells per well. After 2 h incubation at 37°C, cells were overlaid with 2 ml of carboxyl methyl cellulose (CMC) that contained GMEM (2% CMC:GMEM ratio was 2:3, final concentration of FCS was 1.2%). Plaques were stained with crystal violet after 2 days incubation at 37°C. The stocks of replication-competent SFV4 vectors were propagated and assayed for stability of d1EGFP expression as described previously [18,19].

### Analysis of expression of vector-encoded proteins

BHK-21 cells (8× 10^6^) were transfected with 10 μg of infectious transcripts or infectious plasmids. At 12 h post-transfection, cells were lysed with Laemmli buffer and the vector-expressed proteins were subjected to SDS-PAGE in 12% gel (lysate from 50,000 cells was loaded on each column). Proteins were transferred to a nitrocellulose membrane and probed with rabbit polyclonal antiserum against SFV nsP1, rabbit polyclonal antiserum against SFV capsid protein, or rabbit polyclonal antiserum against EGFP (all prepared in-house). Western blots were visualised by use of HRP-conjugated anti-rabbit antibody and ECL Immunoblot Detection Kit (GE Healthcare). d1EGFP expression was detected by fluorescent microscopy and BD LSR II flow cytometry.

### Virus RNA purification and Northern blot analysis

BHK-21 cells (1.5× 10^6^) were infected at an moi of 5 with P1 stocks of replication – competent vectors of SFV4, cells infected SFV4 and mock-infected cells were used as controls. Total RNA was purified with TRIzol reagent (Invitrogen). Equal amounts (10 μg) of RNA were denatured for 10 min at 65°C in formamide-formaldehyde buffer and electrophoretically separated on 1.5% agarose gels supplemented with 0.2 M formaldehyde. The separated RNAs were transferred to Hybond-N+ membrane (Amersham Biosciences) and UV cross-linked. Hybridization with an RNA probe, complementary to the 3'-UTR of SFV4 genome, was performed by standard procedure. The filter was exposed to X-ray film or analyzed by Typhoon TRIO equipment and ImageQuant TL software (GE Healthcare).

## Competing interests

All authors are also inventors in patent application PCT/EE2008/00020 "A method for creating a viral genomic library, viral genomic library and a kit for creating the same", which describes potential industrial applications of several vectors and approaches described in this manuscript; the owner of IP rights is the University of Tartu.

## Authors' contributions

KR carried out the protein expression analysis, and construction and analysis of vector-based expression libraries. AI carried out molecular cloning of viral expression vectors. LÜ analysed the viral RNA synthesis in infected cells. LKA and LÜ analysed the growth of vectors in infected cells. AI, KR and LÜ carried out the vector stability analysis. VL developed the *in vitro *ligation method and constructed corresponding tools. AM conceived the study, participated in its design and coordination, and drafted the manuscript. All authors read and approved the final manuscript.
